# Local delivery of AAV2-CTLA4IgG decreases sialadenitis and improves gland function in the C57BL/6.NOD-*Aec1Aec2 *mouse model of Sjögren's syndrome

**DOI:** 10.1186/ar3753

**Published:** 2012-02-27

**Authors:** Hongen Yin, Cuong Q Nguyen, Yuval Samuni, Toshimitsu Uede, Ammon B Peck, John A Chiorini

**Affiliations:** 1 Molecular Physiology and Therapeutics Branch, National Institute of Dental and Cranial Research, National Institutes of Health, 10 Center Drive, MSC1190, Bethesda, MD 20892, USA; 2 Department of Oral Biology, College of Dentistry, University of Florida, Gainesville, Florida; 3 Section of Immunopathogenesis, Institute of Immunological Science, Hokkaido University, Sapporo, Hokkaido 060, Japan

**Keywords:** Sjögren's Syndrome, salivary gland dysfunction, adeno-associated virus (AAV), CTLA4IgG fusion protein, prevention

## Abstract

**Introduction:**

Cytotoxic T-lymphocyte antigen 4 (CTLA-4) is a key negative costimulatory molecule that displays a wide range of anti-inflammatory properties and is currently approved to treat rheumatoid arthritis as a recombinant fusion protein (CTLA4IgG). To better understand the role of CTLA4IgG in primary Sjögren's syndrome (pSS), we generated a recombinant adeno-associated virus vector serotype 2 (AAV2) expressing a chimera of mouse CTLA-4 fused with a human immunoglobulin (AAV2-CTLA4IgG) and observed the effect of this molecule in C57BL/6.NOD-*Aec1Aec2 *mice, an animal model of pSS.

**Methods:**

A recombinant adeno-associated virus-2 (AAV-2) vector was constructed encoding a CTLA4IgG fusion protein. The AAV2-CTLA4IgG vector and an AAV2 control vector encoding beta galactosidase (LacZ) were administered by retrograde cannulation of the submandibular glands of C57BL/6.NOD-*Aec1Aec2 *mice. Protein expression was measured by ELISA and salivary glands were assessed for inflammation and activity.

**Results:**

Recombinant CTLA4IgG blocked B7 expression on macrophages *in vitro. In vivo*, localized expression of CTLA4IgG in the salivary glands of C57BL/6.NOD-*Aec1Aec2 *mice inhibited the loss of salivary gland activity and decreased T and B cell infiltration as well as dendritic cells and macrophages in the glands compared with control mice. In addition a decrease in several proinflammatory cytokines and an increase in transforming growth factor beta-1 (TGF-β1) expression were also observed.

**Conclusions:**

These data suggest expression of CTLA4IgG in the salivary gland can decrease the inflammation and improve the xerostomia reported in these mice.

## Introduction

Primary Sjögren's syndrome (pSS) is a chronic autoimmune disorder that results in impaired exocrine gland function. Xerostomia (dry mouth) and xerophthalmia (conjunctivitis sicca, dry eyes) are hallmarks of Sjögren's syndrome [1]. The mechanism associated with Sjögren's syndrome is unclear however, immunologically-activated or apoptotic glandular epithelial cells may present novel autoantigens in predisposed individuals driving autoimmune-mediated tissue injury [2,3]. Immune activation is typically presented as focal, mononuclear (T, B, dentritic, and macrophage) cell infiltrates, proximal to the ductal epithelial cells and forms sialadenitis [2]. Lymphocytic infiltrations in the salivary gland (SG) and lachrymal glands (LG) consist of 60% to 70% CD4+ T-lymphocytes, and a substantial numbers of B cells, dendritic cells (DCs), plasma, and macrophage (Mф) cells [2,4,5]. Abnormal activation of proinflammatory Th1 [6,7], and Th17 [8] cells and cytokines such as interferon-γ (IFN-γ), interleukin-12 (IL-12), IL-17 were reported in animal models of pSS and patient samples [7-11]. In addition, a dramatic drop in T regulatory cells (Treg) and decreased expression of TGF-β1 in SG infiltrates was also reported in pSS patients [12]. Furthermore, TGF-β1 deficient mice develop a Sjögren's syndrome like autoimmune disease [13].

Cytotoxic T-lymphocyte antigen 4 (CTLA-4) is an important immune regulatory protein and displays a wide range of activities associated with immune tolerance. By competing with CD28 to bind B7-1/2 (CD80/CD86), CTLA4 blocks the activation of T cells, thus maintaining immune homeostasis [14]. CTLA-4 is constitutively expressed on Treg cells and also binds to B7 on antigen-presenting cells (APCs) to inhibit activation of effector T cells [15,16]. Recently it is noted that epithelial cells in the minor SGs of pSS patients express costimulatory molecules B7.1 (CD80) and B7.2 (CD86) [17]. Correspondingly, different haplotypes of CTLA-4 were found to be associated with increased susceptibility to pSS [18].

Currently, a recombinant fusion protein of CTLA4-immunoglobulin (CTLA4-Ig, Abatacept, Orencia) is licensed in the United States for the treatment of rheumatoid arthritis [5]. Abatacept, which contains the CTLA-4 high-affinity binding site for B7 blocks B7:CD28 costimulatory signaling pathway and is reported to shut down activation of proinflammatory T cells [19] as well as B cells, DCs, and Mф [20,21].

In order to study the affect of CTLA4 blockade on the sialadenitis and xerostomia associated with Sjögren's syndrome, CTLA4IgG was locally expressed in the salivary glands of C57BL/6.NOD-*Aec1Aec2 *mice, which develop a Sjögren's syndrome-like disease. Our findings of both functional and immunological improvement in the mice warrant further investigation of CTLA4 mediated immunomodulation as a therapeutic pathway for treatment of pSS patients.

## Materials and methods

### Cell lines

HEK-293T cells were grown in Dulbecco's modified Eagle's medium (DMEM). Medium was supplemented with 10% heat-inactivated fetal bovine serum (Life Technologies, Rockville, MD, USA), 2 mM L-glutamine, penicillin (100 U/ml), and streptomycin (100 μg/ml; Biofluids, Rockville, MD, USA) as previously described [22].

### Construction and testing of AAV2-CTLA4IgG

We previously reported construction of AAV2-LacZ [23]. In this study we used the extracellular domain of mouse Cytotoxic T-lymphocyte antigen 4 (CTLA4) coupled to human Immunoglobulin G (IgG) Cγ1 (CTLA4IgG), kindly provided by Dr Toshimitsu Uede (Institute of Immunological Science, Hokkaido University, Hokkaido, Japan) [24]. This gene was cloned into the recombinant adeno-associated virus (AAV) plasmid containing a cytomegalovirus (CMV) promoter and the inverted terminal repeat (ITRs) sequences for AAV serotype 2 (AAV2). The plasmid (AAV2-CTLA4IgG) was transfected into HEK-293T cells and expression of the protein in the media was determined by western blotting after electrophoresis in reducing conditions using anti-mCTLA4 Ab (R&D systems, Minneapolis, MN, USA).

### Competitive inhibition of B7 association by CTLA4IgG *in vitro*

Mouse macrophages (CRL-2751, ATCC) were grown in DMEM with 4 mM L-glutamine, 1.5 g/L sodium bicarbonate, 4.5 g/L glucose (Biofluids, Rockville, MD, USA), 10% fetal bovine serum, and 20% LADMAC conditioned media (produced from the LADMAC cell line (CRL-2420) at 37°C in a humidified, 5% CO_2 _atmosphere, incubator. 1 × 10^5 ^cells/well were placed in round bottom 96-well plates and spun down at 1500 rpm in a bench top centrifuge at 4°C. The cells were then washed twice with PBS (pH 7.4, 0.05% Tween 20) and incubated for 1 h at 37°C with either medium from control HEK-293T cells or from HEK-293T cells transfected with AAV2-CTLA4IgG. Following additional washes, the cells were incubated in the dark with 0.5 to 1 μg/ml of Armenian hamster IgG FITC-conjugated anti B7-1 (Santa Cruz Biotechnology, Santa Cruz, CA, USA) in blocking solution (PBS, pH 7.4, 0.5% BSA) at 4°C for 40 min. The cells were then washed and analyzed by flow cytometry assay.

### Vector production

To generate AAV2 vectors, we used the adenoviral helper packaging plasmid pDG. Fifteen-cm plates of approximately 40% confluent HEK-293 T cells were co-transfected with either AAV-LacZ or AAV-CTLA4IgG according to standardized methods [25]. Clarified cell lysates were adjusted to a refractive index of 1.372 by addition of CsCl, and centrifuged at 38,000 rpm for 65 h at 20°C. Equilibrium density gradients were fractionated and fractions with a refractive index of 1.369 to 1.375 were collected. The particle titer was determined by Q-PCR and the vector was stored at -80°C. On the day of vector administration to C57BL/6.NOD-*Aec1Aec2 *mice, the vector was dialyzed for 3 h against saline.

### Animals

Three female and ten male C57BL/6.NOD-*Aec1Aec2 *mice, aged six weeks, were bred and maintained at the animal facility of the Department of Pathology, University of Florida, as described previously [26]. Baseline saliva and tear flow was collected from mice when they were six weeks old. Gene therapy studies, as described herein, were approved by the University of Florida's IACUC and IBC.

### AAV2 vector administration

Mice were randomly grouped and vectors were delivered into the submandibular glands by retrograde instillation as previously described [22] (AAV2-LaZ: 1 female, 5 males and AAV2-CTLA4IgG: 2 females, 5 males). As previously reported, the vector was well tolerated with no vector related inflammation [22,27]. Briefly, eight-week-old mice were mildly anesthesized with ketamine (100 mg/ml, 1 ml/kg body weight; Fort Dodge Animal Health, Fort Dodge, IA, USA) and xylazine (20 mg/ml, 0.7 ml/kg body weight; Phoenix Scientific, St Joseph, MO, USA) solution given by intramuscular injection (IM). Ten minutes after IM injection of atropine (0.5 mg/kg BW; Sigma, St Louis, MO, USA), mice were administered 50 μl AAV2 vector into both submandibular glands by retrograde ductal instillation (1 × 10^10 ^particles/gland) using a thin cannula.

### Detection of CTLA4IgG expression in salivary glands and serum from C57BL/6.NOD-*Aec1Aec2 *mice

To confirm the stable expression of CTLA4IgG *in vivo *after local delivery in the SGs from C57BL/6.NOD-*Aec1Aec2 *mice, homogenates of SGs were prepared as described previously [7]. Total protein in the supernatant was determined with BCA™ protein assay kit (Pierce, Rockford, IL, USA) according to the manufacturer's instructions. Blood was collected by cardiac puncture at the time of sacrificing and collected in microcentrifuge tubes. Serum was separated by centrifugation for 20 min at 2000 g and stored at -80°C.

CTLA4IgG was detected by sandwich ELISA. A 96-well plate (Nunc, Rochester, NY, USA) was incubated overnight (O/N) with 0.4 μg/ml capture antibody, goat anti-mouse CTLA-4 Ab (R&D, Minneapolis, MN, USA) in carbonate/bicarbonate buffer (pH9.5). The next day, wells were blocked and incubated with 100 μL of appropriately diluted standard control (0.0850 μg/ml rCTLA4, R&D, Minneapolis, MN, USA) or serum, undiluted SG homogenates for 2 h according to the product instructions. Following three washings, the wells were incubated with a 1:5,000 dilution of peroxidase conjugated affinity purified goat anti-human IgG (Jackson ImmunoResearch, West Grove, PA, USA) for 1 h at RT. Substrate reaction was developed following the manufacturer's protocol and the plate were read at OD 450 nm using a Microplate reader model 680 (Bio-Rad Laboratories, Hercules, CA, USA) [7].

### Measurement of salivary and tear flow rates

Pilocarpine stimulated saliva flow rate (SFR) and tear flow rate (TFR) collection was performed as described previously [8] at the indicated time points. Briefly, individual non-anesthetized mice were weighed and given an i.p. injection of 100 μl of PBS containing a mixture of isoproterenol (0.02 mg/ml) and pilocarpine (0.05 mg/ml). Saliva was collected from the oral cavity of individual mice for 10 min. At week 30, tear volumes from individual animal were measured after i.p. injection of pilocarpine (4.5 mg/g [BW]) for 20 s and determined using a phenol red thread (Zone-Quick, FCI Ophthalmics, Pembrooke, MA, USA), a modified version of the Schirmer test as described [8]. SFR and TFR were then calculated per gram body weight.

### Determination of autoantibodies

At the end of the study, serum was collected from 30-week-old C57BL/6.NOD-*Aec1Aec2 *mice. An ELISA was developed as described previously [7] to detect autoantibodies against SSA/Ro [multiple antigenic peptide (MAP)-Ro273] (University of Oklahoma Health Sciences Molecular Biology core Facility, Oklahoma City, OK, USA). Autoantibodies against SSB/La (total Ig) were measured by a commercially available ELISA kit (Alpha Diagnostic International, San Antonio, TX, USA) according to the manufacturer's protocol.

### Histological assessment of salivary glands

Following euthanasia, whole SGs were surgically removed from each mouse and placed in 10% phosphate-buffered formalin for 24 h for hematoxylin and eosin (H&E) staining as described previously [10]. Stained sections were observed under a microscope for glandular structure and leukocyte infiltration. Unstained sections were used for immunofluorescence and immunochemistry staining (below).

### Immunofluorescence staining for CD3+T cells and B220+B cells

Immunofluorescence staining for T and B cells for the infiltrations in the SGs was done as previously described [8]. Briefly histological sections of salivary glands were incubated with rat anti-mouse B220 (BD Pharmingen, San Jose, CA, USA) and goat anti-mouse CD3 (Santa Cruz Biotechnology, Santa Cruz, CA, USA), followed by incubation with Texas Red-conjugated rabbit anti-rat IgG (Biomeda, Foster City, CA, USA) and FITC-conjugated rabbit anti-goat IgG (Sigma-Aldrich, St. Louis, MO, USA). The slides were mounted with DAPI-mounting medium (Vector Laboratories, Burlingame, CA, USA). Sections were observed at 200× magnification using a Zeiss Axiovert 200M microscope, and images were obtained with AxioVs40 software (Ver. 4.7.1.0, Zeiss) (Carl Zeiss, Thornwood). The number of lymphocytic foci (LF) in each section was blindly enumerated by three individual investigators. The number of B and T cells, and total number of nuclei in each LF were determined using Mayachitra image software (Mayachitra, Inc., Santa Barbara, CA, USA).

### Immunohistochemical staining for CD11c and F4/80 in salivary glands

Paraffin-embedded SGs were deparaffinized by immersion in xylene, followed by antigen retrieval with 10 mM citrate buffer, pH 6.0. Tissue sections were then incubated overnight at 4°C with anti-CD11c or anti-F4/80 antibody (Santa Cruz Biotechnology Santa Cruz, CA, USA). Total rabbit IgG was used as an isotype control. The slides were incubated with biotinylated goat anti-rabbit IgG secondary antibody followed by horseradish peroxidase-conjugated strepavidin incubation using the Vectastain ABC kit. The staining was developed by using diaminobenzidine substrate (Vector Laboratories, Burlingame, CA, USA), and counterstaining was performed with hematoxylin. Sections were photographed at 200× magnification using a Zeiss Axiovert 200M microscope, and images were obtained with AxioVs40 software (Ver. 4.7.1.0, Zeiss) (Carl Zeiss, Thornwood). The number of CD11c or F4/80-positive cells was counted by three different examiners and the mean of three counting were determined.

### Detection of cytokines from immune cell cultures and SG homogenates

Cytokines from spleen cell and draining lymph nodes (DLN) cell culture, serum, and homogenates of SGs were detected as described previously [28]. Briefly cells from spleen and submadibular salivary gland (SMGs) associated DLNs obtained from treated mice were isolated respectively and samples from mice in each group were pooled and cultured in 24-well plates at 5 × 10^6 ^cells/ml RPMI-1640 medium (Invitrogen, Carlsbad, CA, USA), containing HL-1 serum replacement (Cambrex Bioscience, Walkersville, MD, USA), with or without 1 μg/ml Concanavalin A (ConA, Sigma-Aldrich, St Louis, MO, USA). Supernatants were collected after 48 h incubation. Serum and SMG homogenates were prepared as described previously [7,28] and three samples from representative mice in each group were used for detection.

Murine interleukin-6 (IL-6), tumor necrosis factor-α (TNF-α), IL-12 p70, interferon-γ (IFN-γ), IL-18, IL-17, IL-23, transforming growth factor-β1 (TGF-β1), mast cell proteinase-1 (MCP-1), and macrophage inflammatory proteins-1 (MIP-1) were measured using a multiplex sandwich-ELISA assay (Aushon Biosystem Billerica, MA, USA). Duplicates for each sample were tested in three dilutions and the mean values of the duplicates from the optimal dilution were reported [28].

### Statistical analysis

Mann-Whitney U test was used to analyze differences of cytokine production in serum. Unpaired student t-test was used to analyze differences between two experimental groups in other assays. All analyses were performed with GraphPad Prism statistical software (GraphPad Software Inc. version 4.02, La Jolla, CA, USA) using a *P *value ≤0.05 as statistically significant.

## Results

### Detection of CTLA4IgG and binding to B7 *in vitro*

Prior to analyzing stable expression of CTLA4IgG from AAV2-CTLA4IgG in the SGs of C57BL/6.NOD-*Aec1Aec2 *mice, we confirmed both the expression and the biological activity of the fusion protein by western blot, and by blocking the B7:CD28 pathway *in vitro*, respectively.

**Figure 1:**
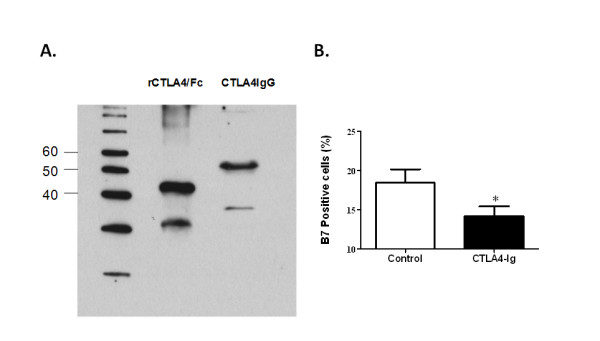
***In vitro *expression and activity of CTLA4IgG**. **(A) ***In vitro *expression of CTLA4IgG was detected by western blot of media from AAV2-CTLA4 transfected cells (lane 3). As a control, purified recombinant mouse CTLA4/Fc was also run on the gel (lane 2). **(B) **Biological activity of CTLA4IgG, based on its ability to bind and block B7.1, was determined by incubating with either medium from naïve HEK-293T cells (column 1) or from cells transfected with AAV2CTLA4 (column 2). Unbound B7.1 was then tested by flow cytometry using an antibody to B7.1. Data shown is mean from three independent experiments (asterisk, *P *= 0.0400). Unpaired student-t test was used in this analysis.

Fusion of the Cγ1 domain of IgG to the binding domain of CTLA4 resulted in a chimeric protein of approximately 62 kDa. Secretion was confirmed by western blot of the cell culture media from transfected cells (Figure 1A).

Macrophages, like dendritic cells, are one of the antigen presenting cells (APCs) that express the costimulatory molecule B7. To test the ability of the recombinant CTLA4IgG to bind and block B7 detection, supernatant from CTLA4IgG expressing cells was preincubated with macrophages and then B7 quantified by flow cytometry assay. Compared to macrophages incubated without CTLA4IgG, unbound and detectable B7 expression on the macrophages incubated with CTLA4IgG was decreased, suggesting binding between B7 and CTLA4IgG.

### *In vivo *expression of CTLA4IgG following cannulation with AAV2-CTLA4IgG in C57BL/6.NOD-*Aec1Aec2 *mice

**Figure 2:**
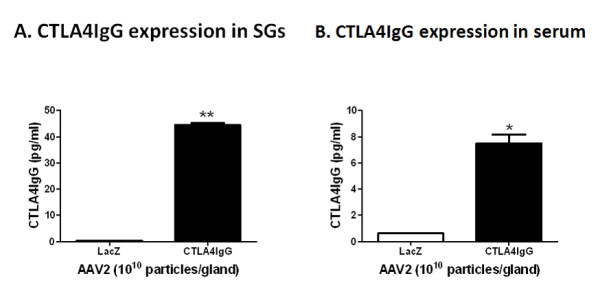
***In vivo *expression of CTLA4IgG in salivary glands from C57BL/6.NOD-*Aec1Aec2 *mice**. A sandwich ELISA was developed to detect expression of mouse CTLA4 and human IgG (CTLA4IgG) chimeras in homogenates of submandibular salivary glands (**A**) and serum (**B**) (Materials & Methods). Data show the mean ± SEM from each group. Mice cannnulated with AAV2-CTLA4IgG (*n *= 6, pooled into two samples/group) had significant levels of CTLA4IgG protein in the salivary glands compared with mice that received AAV2-LacZ (*n *= 7, pooled into two samples/group), (two asterisks, *P *= 0.0003) and serum (asterisk, *P *= 0.0102) Unpaired student-t test was used in this analysis.

Salivary gland infiltrating lymphocytes in C57BL/6.NOD-*Aec1Aec2 *are first detected between 8 and 12 weeks of age. However, microarray analysis indicates that adhesion molecules as well as genes associated with macrophages and dendritic cells are upregulation as early as 8 weeks of age. Prior to 8 weeks SGs are not considered fully mature. Thus, we chose to cannulate the glands at 8 weeks which would be considered an our early stage of disease [29]. As previously reported, cannulation of these mice with AAV vectors was well tolerated and no vector related inflammation was observed [22,27]. To confirm the stable expression of CTLA4IgG *in vivo *after local delivery of AAV2-CTLA4IgG to the SGs of C57BL/6.NOD-*Aec1Aec2 *mice, SG homogenates and serum were obtained at the end of the study (30 weeks) and pooled according to each group. Using a sandwich-ELISA to detect the recombinant chimeric protein (mouse CTLA4 and human IgG), mice that received AAV2-CTLA4IgG had much higher levels of CTLA4IgG (44.5 ± 0.76 pg/ml in SG and 7.48 ± 0.70 pg/ml in serum, mean ± SD) in both their SG and serum compared with mice that received vector expressing LacZ (0.39 ± 0.02 pg/ml in SG and 0.62 ± 0.01 pg/ml in serum) (*P *= 0.0003 and *P *= 0.0102, respectively, in SG and serum) (Figure 2). In agreement with the local delivery of vector, the level of expression in the SG homogenates was elevated compared with serum in the AAV2- CTLA4IgG-treated mice.

### Local expression of AAV2-CTLA4IgG prevents the age-dependent loss of salivary gland activity in C57BL/6.NOD-*Aec1Aec2 *mice

**Figure 3:**
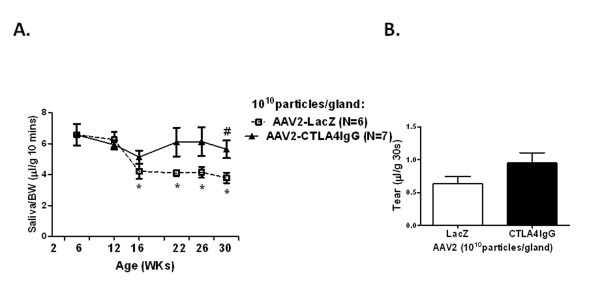
**Stimulated saliva and tear flow in treated C57BL/6.NOD-*Aec1Aec2 *mice**. Saliva and tear flow were collected as described in the Materials & Methods section. The data shown represent the mean ± SEM flow per group (*n *= 6 in AAV2-LacZ group and *n *= 7 in AAV2-CTLA4IgG group). Unpaired student t-test was used in this analysis. **(A) **Mice treated with vector expressing CTLA4IgG showed protection from loss of gland activity. Saliva was collected over a 10-min period after stimulation with a 100 μl of PBS containing a mixture of isoproterenol (0.02 mg/ml) and pilocarpine (0.05 mg/ml) and tear flow were collected over a 20-s period after injection of pilocarpine (4.5mg/kg body weight). AAV2-LacZ mice showed decreased saliva on weeks 16, 22, 26, and 30 (asterisk, *P *= 0.0428, 0.0217, 0.0292, and 0.0128, respectively), compared with the baseline saliva collection on week 6 (*n *= 9, 5.933 ± 0.2969). AAV2-CTLA4IgG-treated mice had a slight decrease of saliva but this was not significant at 16 weeks (*P *= 0.2057). Saliva flow of CTLA4IgG-treated mice increased to baseline level by 22 weeks (6.13 ± 0.92 μL/g 10 min). CTLA4IgG-treated mice had increased saliva flow compared with LacZ-treated mice by 30 weeks (*P *= 0.0232). **(B) **Delivery of AAV2-CTLA4IgG resulted in an increase in tear flow (mean ± SEM) by 30 weeks compared with control mice, but this was not significant (*P *= 0.1316).

To better understand the effect of CTLA4IgG on SG function, stimulated saliva flow was measured in both treated and control mice over time. In agreement with previous studies [26], mice treated with the control LacZ expressing vector showed a significant decrease of saliva flow by 16 weeks (4.25 ± 0.64 μL/g 10 min), compared with the baseline flow at 6 weeks (6.10 ± 0.30 μL/g 10 min), which continued to decline over time [8]. Mice that received AAV2CTLA4IgG vector initially also showed some decrease in saliva flow at 16 weeks (5.13 ± 1.22 μL/g 10 min), but it was not statistically significant compared with the 6-week baseline (*P *= 0.2057). However, by 22 weeks the saliva flow from the CTLA4IgG mice had recovered to near baseline values (6.13 ± 0.92 μL/g 10 min), which was sustained for the remainder of the study, and was statistically different compared with the AAV2-LacZ-treated group at 30 weeks (*P *= 0.0222). These data suggest that expression of CTLA4IgG in the SGs of C57BL/6.NOD-*Aec1Aec2 *mice can inhibit the loss of SG function (Figure 3A).

### Salivary gland transduction with AAV2 CTLA4IgG had only minimal affect on lachrymal gland dysfunction in C57BL/6.NOD-*Aec1Aec2 *mice

Previous research has suggested that following cannulation of the SG, greater than 90% of the AAV2 vector remains in the gland [27]. Similarly higher levels of the expression can also be detected locally in the gland homogenates compared with circulating levels. In order to test if the circulating levels of CTLA4IgG were sufficient to have an effect on other secretory epithelia such as the loss of lachrymal gland function in the C57BL/6.NOD-*Aec1Aec2 *mice, we measured stimulated tear flow at the end of the study. CTLA4IgG-treated mice had an overall increase in tear flow compared with the LacZ-expressing group, but was not significantly different (*P *= 0.1316) (Figure 3B).

### CTLA4IgG expression does not change autoantibody levels in C57BL/6.NOD-*Aec1Aec2 *mice

**Figure 4:**
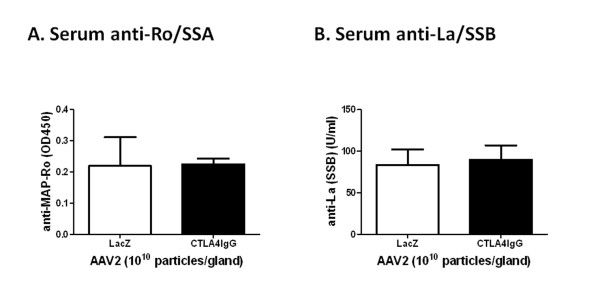
**Serum anti-nuclear antibody productions in C57BL/6.NOD-*Aec1Aec2 *mice**. Serum samples were analyzed for anti-Ro (SSA) **(A) **and anti-La (SSB) **(B) **antibody expression in serum from AAV2-LacZ (*n *= 6) and AAV2-CTLA4IgG (*n *= 7) treated mice by ELISA. The data shown represent the mean ± SEM in OD or U/ml from duplicate tests of pooled samples from each group. Unpaired student's t-test was used for statistical analysis. No statistically significant difference was detected (*P *= 0.9586 and 0.4158, respectively).

To observe the effect of CTLA4IgG on the regulation of systemic B cell activation, we measured anti-Ro (SSA) and anti-La (SSB) autoantibodies, which are highly correlated with pSS [30]. Minimal changes in anti-Ro and anti-La titer were detected in the CLTA4IgG-treated mice compared with the AAV2-LacZ-treated mice (Figures 4A and 4B).

### Salivary gland transduction with AAV2 CTLA4IgG can decrease infiltrating T and B cells, DCs, and Mф

**Figure 5:**
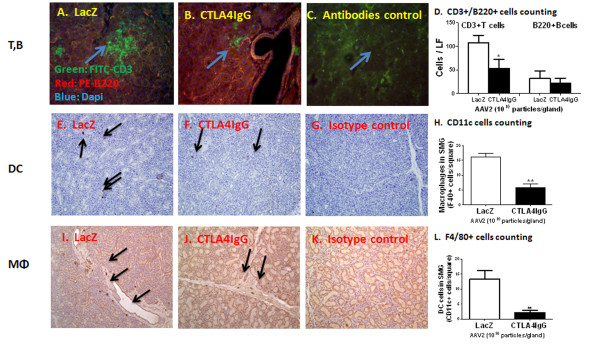
**Histological examination of salivary glands**. Salivary gland histology was examined at the end of the study (30 weeks of age). CD3+T and B220+B cell immunofluorescence staining, as well as CD11c and F4/80 immunochemistry staining for dendritic and macrophages cells respectively were done as described in Materials and Methods. Panels show representative immuno-fluorescence staining of salivary of B and T cells in SGs from mice cannulated with AAV2-LacZ (*n *= 6) or AAV2-CTLA4IgG vector (*n *= 7) **(A, B, and C) **(Blue arrows) and enumeration (mean ± SEM) **(D) **of B and T cells in SGs from LacZ- and CTLA4IgG- treated mice; immunohistochemical staining and enumeration (mean ± SEM) of CD11c+ DCs **(E, F, G, and H) **and F4/80+ macrophages (**I, J, K, and L) **(Black arrows) in SGs from LacZ- and CTLA4IgG-treated C57BL/6.NOD-*Aec1Aec2 *mice. A statistical decrease in the enumeration of both T cells was shown in the SGs from CTLA4 overexpressing mice compared to the LacZ-treated (*P *= 0.0464). No statistical significance but a trend was shown (*P *= 0.3024) for decrease trend of B cells. Significant down-regulation of both CD11c+ dendritic cells and F4/80+macrophages was seen in the SGs from CTLA4IgG-treated mice compared with control (two asterisks, *P *≤0.01). Unpaired student-t test was used in the analysis.

To determine the effect of CTLA4IgG on lymphocyte foci (LF) in the SGs, we detected the number of LF as well as the number of T and B cells within the gland by immunofluorescence staining of CD3 and B220, respectively (Figures 5A, B, C, and 5D). The number of LF were decreased in the SGs of CTLA4IgG mice (0.71 LF/per gland) compared with control LacZ mice (2.16 LFs/per gland). Furthermore, the number of T and B cells present in the LF also decreased. Although the decrease in T cells was significant, the change in B cells was not statistically significant compared with control LacZ-treated mice (*P *= 0.0464 and *P *= 0.3024 for analysis of T and B cells, respectively) (Figures 5C, D, E and 5F).

An increase in DCs and macrophages has also been reported in the SGs of pSS patients [31]. The number of DCs and Mф cells (detected by staining for CD11c+ DCs and F4/80+, respectively) was significantly decreased in the CTLA4IgG-treated group compared with the LacZ control group (DCs: 13.33 ± 2.78/gland in LacZ group *vs*. 2.14 ± 0.70/per gland in CTLA4IgG mice, *P *≤0.01. Mф: 14.89 ± 0.11/gland in LacZ group *vs*. 3.84 ± 2.31/per gland in CTLA4IgG mice, *P *≤0.0001) (Figure 5E, F, G, H, I, J, K and 5L). These data indicate that local expression of CTLA4IgG can inhibit T-cell accumulation in the SG, as well as DCs and Mф.

### CTLA4IgG expression decreased proinflammtory cytokines and increased TGF-β1 in salivary gland associated lymph nodes

To investigate cytokine production after CTLA4IgG expression, we measured the levels of cytokine associated with different populations of T cells or macrophages in both spleen and serum as well as locally in the SG and associated draining lymph nodes (DLN).

In SG homogenates, only a decrease in IL-6 was observed in the AAV2-CTLA4IgG-treated mice (median = 169.60 pg/ml) compared with AAV2-LacZ controls (median = 86.51 pg/ml), which was not statistically significant (*P *= 0.9062, data not shown). Interestingly, TGF-β1 production increased in the CTLA4IgG group compared with control LacZ-treated mice, however the increase was not statistically significant (median = 1208.70 pg/ml *vs*. median = 804.53 pg/ml, respectively) (*P *= 0.093, data not shown). In the pooled cell culture media of the DLN cells associated with the SG, a general down-regulation in proinflammatory cytokine was observed in the CTLA-4IgG-treated mice compared with control LacZ mice. Th1 cytokines (IL-12, IFN-γ, and IL-18) and Th17 cytokine (IL-23) were all down-regulated. Again, TGF-β1 was strikingly up-regulated. In addition, non-specific proinflammatory cytokines, IL-6 and TNF-α, as well as chemokines MCP-1 and MIP-1α, which are mainly released from macrophages, were decreased (Table 1). Little change was detected in Th2 cytokines such as IL-4, IL-5, and IL-13 after local expression of CTLA4IgG (data not shown). These changes suggest that CTLA4IgG expression can reduce proinflammatory cytokines released by Th1, Th17 cells, DCs, and macrophages, while stimulating production of anti-inflammatory cytokines such as TGF-β1. This further supports the finding of reduced inflammation with CTLA4IgG treatment.

**Table 1:** Cytokine production local and systemic immune system in vector treated mice (pg/ml)

		DLN cells		Spleen cells		Serum	
		
		LacZ	CTLA4IgG	LacZ	CTLA4IgG	LacZ	CTLA4IgG
Th1-	IL-12p70	12	0.00↓	4.6	0.20	88.5 ± 15.8	88.5 ± 15.8
	IFN-γ	3.8	0.00↓	122	115.0	872.6 ± 1,276.8	457.2 ± 371.8↓
	IL-18	11	0.00↓	16	0.00↓	694 ± 562	394.10↓
Th17-	IL-17	N/A	N/A	2.1	0.00↓	2.8 ± 3.3	N/A↓
	IL-23	14	N/A↓	78.2	36.10↓	335.6 ± 365.7	101.99↓
Treg- or SG epithelial cells	TGF-β1	N/A	41,410↑	2,084	1,384	1,501,176 ± 277,249	1,260,620 ± 289,478
Non-specific (Macrophages)	IL-6	2452	1,688↓	2,557	2,407	309 ± 1,241	289 ± 355
	TNF-α	43	27↓	98	120	37 ± 83	41 ± 142
Chemokines	MCP-1	10	2↓	44	43	63 ± 19	66 ± 26
	MIP-1α	224	130↓	357	237	2 ± 0.2	2.5 ± 2.5

Serum, splenocytes, and salivary gland DLNs were collected at the end of the study. Splenocytes and DLN cells were pooled according to vector treatment group. Culture supernatants were collected following incubation with or without ConA for 48 h. Cultures and serum were then analyzed for levels of the indicated cytokines (in pg/ml) by multi-cytokine assay in duplicate. For cell cultures, values are the mean of duplicate tests of ConA treated cells subtracted from media alone. For serum, the data shown were the median ± SD of each group of mice (AAV2-LacZ [*n *= 6] and AAV2-CTLA4IgG [*n *= 7], respectively]. Mann-Whitney U test was used to analyze differences of cytokine production in serum. ↑: Production of cytokines in the AAV2-CTLA4IgG group is ≥ 50% higher than in AAV2-LacZ group; ↓: Production of cytokines in the AAV2-CTLA4IgG group is ≥ 50% lower than the AAV2-LacZ group.

### Effect of CTLA4IgG on regulation of systemic T cell response in the C57BL/6.NOD-*Aec1Aec2 *mice

To test for changes in the systemic immune system, cytokines were also measured in serum and in spleen cell cultures. Wide variation was seen in cytokine values in serum, and none of the cytokine levels from the CTLA4IgG and LacZ groups showed statistical significance. However, a decrease (a change in the median of ≥ 50%) in a majority of cytokines associated with Th1 and Th17 cells, such as IFN-γ, IL-18, IL-17, and IL-23, was seen in the serum of AAV2-CTLA4IgG-treated mice compared with AAV2-LacZ-treated mice. In addition, production of IL-12, IL-18, IL-17, and IL-23 from splenocytes was down-regulated in the AAV2-CTLA4IgG-treated group compared with the AAV2-LacZ-treated mice. Unlike the local immune response seen in the SG associated DLN, minimal change in non-specific cytokines, chemokines, or TGF-β1 was detected in serum or cultured splenocytes. These data imply that CTLA4IgG expression can also decrease proinflammatory cytokines in the peripheral immune system, following local SG gene transfer in C57BL/6.NOD-*Aec1Aec2 *mice (Table 1).

## Discussion

The blockade of CD28 co-stimulation by recombinant CTLA4IgG (abatacept) has demonstrated clinical utility in the treatment of rheumatoid arthritis [14]. In our study, local expression of CTLA4IgG by gene transfer to the SGs of C57BL/6.NOD-*Aec1Aec2 *mice, a pSS animal model, resulted in a decrease in the sialoadenitis and improvement in gland function compared with mice that received a control vector.

The advantage of localized gene transfer is to direct the expression of the therapeutic molecule to the site of maximum effect while minimizing the systemic complications that can be associated with off target effects. Using this approach we were able to achieve much higher local concentrations of CTLA4IgG in the salivary glands compared to circulating levels in the serum. Our data further confirm that ductal cells within the gland represent a good depot site for production of recombinant proteins [32]. Indeed, previous experiments have demonstrated expression from salivary gland ductal cells for the life of the animal [33].

In both patients and C57BL/6.NOD-*Aec1Aec2 *mice, activated CD4+ T lymphocytes including Th1 and Th17 cells infiltrate the salivary and lachrymal glands, and produce a variety of proinflammatory cytokines, such as IFN-γ and IL-17, which may trigger gland damage and represent a crucial element in the pathogenesis of pSS [2,34]. Although not statistically significant, we did detect a decrease in Th17 cytokine in both the DLN and spleen following expression of CTLA4IgG, suggesting a corrective shift in this critical cell population.

Besides the negative effect on T cells as a result of blockade of the B7:CD28 costimulatory pathway [19], it is also noted that recombinant CTLA4IgG may directly or indirectly deactivate DCs, macrophages and B lymphocytes [15,21]. Our data indicate that in C57BL/6.NOD-*Aec1Aec2 *mice, CTLA4IgG expression results in a decrease in T and B lymphocytes as well as DCs and macrophages in the SG that is accompanied by a down-regulation in proinflammatory cytokines. Our finding is in agreement with previous reports on the effect of CTLA4IgG in other autoimmue disease model [20,21].

Interestingly, a significant increase in TGF-β1 expression in both the SG and the DLNs was observed. The increase in TGF-β1 expression maybe related to an increase in nTreg or negative regulation of epithelial cells by CTLA4IgG [15]. In addition to its role in the immune system, TGF-β1 expression was found to be important in maintaining epithelial tight junctions, an important component in the fluid movement of SGs [35] and therefore may be directly related to the improvement in saliva flow.

Our study suggests an improvement of SG function, which could result from the inhibition of sialadenitis after local expression of CTLA4IgG. This suggests that local delivery of AAV2-CTLA4IgG is a promising treatment of pSS. In addition, some improvement in lachrymal gland was also observed. This difference is likely related to the lower circulating levels of CTLA4IgG in the serum compared with the levels in the salivary gland. Despite the positive results achieved in this study, the circulating levels of CTLA4IgG are well below those clinically used with abatacept (http://packageinserts.bms.com/pi/pi_orencia.pdf). Further increases in the dose of vector or the use of vectors with improved gene transfer activity in the salivary gland are likely to result in higher circulating levels and may have a more significant impact on extraglandular manifestations of Sjögren's syndrome.

Gene therapy is still considered an experimental procedure. However, to date more than 200 clinical trials have been conducted and recently many are showing promising results in treating hemophilia, cancer, immunodeficiencies, and blindness. An ongoing trial targeting the salivary glands is also reporting positive results suggesting that gene transfer to the salivary gland is possible [36]. In summary, our data suggest that inhibition of the costimulatory pathway CD28 by expression of CTLA4IgG locally in the salivary gland can be a useful approach for reducing the inflammation and improving the secretory activity associated with Sjögren's syndrome.

## Conclusions

Our data suggest expression of CTLA4IgG can reduce the sialadenitis and improve secretory activity in a mouse model of pSS. In addition to identifying a novel intervention in pSS, our findings support the importance of co-stimulatory pathways as a therapeutic target of the disease.

## Abbreviations

AAV2: adeno-associated virus vector serotype 2; ANA: antinuclear antibodies; APC: antigen-presenting cell; CMV: cytomegalovirus; CTLA-4: Cytotoxic T-lymphocyte antigen 4; CTLA4-IgG: CTLA4-immunoglublin; DMEM: Dulbecco's modified Eagle's medium; IFN-γ: Interferon-γ; IL: interleukin; IM: intramuscular injection; ITRs: inverted terminal repeat; LF: lymphocyte foci; LG: lachrymal glands; MAP: multiple antigenic peptide; MCP-1: mast cell proteinase-1; MIP-1: macrophage inflammatory proteins-1; pSS: primary Sjögren's syndrome; SG: salivary gland; TGF-β1: transforming growth factor-β1; TNF-α: tumor necrosis factor-alpha; Treg: T regulatory cells.

## Competing interests

This work was supported by an NIH NIDCR intramural research grant to JAC. None of the authors have a financial disclosure.

## Authors' contributions

All authors were involved in drafting the article, revising it, and made important intellectual contributions. All authors approved the final version. Dr Chiorini had full access to all the data in the study and takes responsibility for the integrity of the data and accuracy of the data analysis. HY, CQN, ABP, and JAC were responsible for the study concept and design. HY, CQN, and YS were responsible for data acquisition. HY, CQN, YS, and JAC were responsible for the analysis and interpretation of the data.
